# DNA barcoding of the *Lemnaceae*, a family of aquatic monocots

**DOI:** 10.1186/1471-2229-10-205

**Published:** 2010-09-16

**Authors:** Wenqin Wang, Yongrui Wu, Yiheng Yan, Marina Ermakova, Randall Kerstetter, Joachim Messing

**Affiliations:** 1 Waksman Institute of Microbiology, Rutgers University, 190 Frelinghuysen Road, Piscataway, NJ 08854, USA

## Abstract

**Background:**

Members of the aquatic monocot family *Lemnaceae *(commonly called duckweeds) represent the smallest and fastest growing flowering plants. Their highly reduced morphology and infrequent flowering result in a dearth of characters for distinguishing between the nearly 38 species that exhibit these tiny, closely-related and often morphologically similar features within the same family of plants.

**Results:**

We developed a simple and rapid DNA-based molecular identification system for the *Lemnaceae *based on sequence polymorphisms. We compared the barcoding potential of the seven plastid-markers proposed by the CBOL (Consortium for the Barcode of Life) plant-working group to discriminate species within the land plants in 97 accessions representing 31 species from the family of *Lemnaceae*. A *Lemnaceae*-specific set of PCR and sequencing primers were designed for four plastid coding genes (*rpoB, rpoC1, rbcL *and *matK*) and three noncoding spacers (*atpF-atpH, psbK-psbI *and *trnH-psbA*) based on the *Lemna minor *chloroplast genome sequence. We assessed the ease of amplification and sequencing for these markers, examined the extent of the barcoding gap between intra- and inter-specific variation by pairwise distances, evaluated successful identifications based on direct sequence comparison of the "best close match" and the construction of a phylogenetic tree.

**Conclusions:**

Based on its reliable amplification, straightforward sequence alignment, and rates of DNA variation between species and within species, we propose that the *atpF-atpH *noncoding spacer could serve as a universal DNA barcoding marker for species-level identification of duckweeds.

## Background

The cost of DNA purification and sequencing has dropped considerably in recent years so that identification of individual species by DNA barcoding has become an independent, subtler method than solely morphological-based classification to distinguish closely related species, which also defines the systematic relationships by analysis of genetic distance. The key element for a robust barcode is a suitable threshold between inter- and intra-specific genetic distances. Sequence variation between species has to be high enough to tell them apart while the distances within species must be low enough for them to cluster together [1]. The mitochondrial coxidase subunit I (*COI*) gene has proven to be a reliable, cost-effective, and easily recovered barcode marker to successfully identify animal species [2-4], but its application in the plant kingdom is impeded by a slow nucleotide substitution rate, which is insufficient for the diagnosis of individual species [5,6]. However, the Consortium for the Barcode of Life (CBOL) plant-working group recently proposed seven leading candidate sequences for use as barcoding markers [7]. Four plastid coding genes (*rpoB, rpoC1, rbcL *and *matK*) and three noncoding spacers (*atpF-atpH*, *psbK-psbI *and *trnH-psbA*) have been selected based on previous investigations among different plant families [8-10]. However, the utility of each of these sequences for individual families of species within the plant kingdom is hardly predictable [11,12].

Although there have been attempts to use the single-locus of *matK *[8], a combination of two loci, *rbcL *and *trnH-psbA *[9], and even multi-loci combinations [13] as barcoding sequences, the use of a unified barcode for the identification of all the land plants would be difficult due to conflicting needs of different researchers. For example, an optimal barcode marker that has been determined empirically to distinguish plants at the family level may prove less useful for making accurate species level identifications. Most of the proposed plant barcode markers were designed primarily for identifying distantly related organisms in biodiversity hotspots such as Panama [14] and Kruger National Park in South Africa [8]. So far, little attention and only a few studies have been devoted to developing unified barcodes suitable for making identifications within a family, within a genus, or between closely related sister species. A test of seven other candidate barcoding sequences in the family of *Myristicaceae *was applied to eight species within a genus and yielded two suitable barcodes [15]. Recently, it has been shown that all three markers (*rbcL*, *trnH-psbA *and *matK*) can discriminate 4 sister species of *Acacia *across three continents [16]. The marker *matK *has been reported to distinguish 5 *Dendrobium *species [17]. More complex approaches have been developed at the subfamily level identification of larger groups of related plants [18]. Although an extensive barcode study for 31

*Carex *species suggested that a single locus or even multiple loci cannot provide a resolution of greater than 60%, it did not include some of the new markers (*atpF-atpH *and *psbK-psbI*) [19]. When *atpF-atpH *and *psbK-psbI *were included for distinguishing *Carex *and *Kobresia*, it could be shown that *matK *identifies 95% as single-locus or 100% of the species when combined with another marker. However, this study used material from a well defined regional perspective, the Canadian Arctic Archipelago, where the number of co-existing closely related species is limited [20]. Our objective was to determine whether one or more of the markers proposed by the CBOL plant-working group would serve as an optimal marker for species-level identification within the family *Lemnaceae*.

The members of the family *Lemnaceae*, commonly called duckweeds, comprise 38 species in five genera [21]. They are all aquatic plants that grow on or below the surface of the water all over the world and they include the smallest flowering plants [22]. They are ideal material for physiological, biochemical, and genomic studies because of their direct contact with medium, rapid growth and relatively small genome sizes [22]. They are valuable means for biomanufacturing through genetic engineering technology and due to the recent progress towards duckweed-based commercial products [23]. They can be easily maintained by vegetative reproduction in aseptic cultivation for decades [23]. The small size of the plant is ideal for maintaining diverse accessions and therefore for evolutionary studies at the DNA level. Some species, such as *Lemna minor*, are used by the Environmental Protection Agency for measuring water quality because their growth rates are sensitive to a wide range of environmental contaminants such as metals, nitrates, and phosphates [24]. Indeed, wastewater treatment with duckweed has been proposed as a "green" way to remediate municipal water supplies [25]. Rapid growth also offers practical applications of duckweeds as a biofuel crop. Some duckweeds form starch-rich over-wintering fronds called turions, which can be easily induced from vegetative fronds by treatment of cold shock, starvation, or with abscisic acid [26,27]. Resulting from their size and density, both vegetative fronds and turions are much more easily harvested than microalgae [28], which make duckweeds an attractive feedstock for bioethanol production that does not compete for agriculturally productive land.

Given these potential uses, the 160-Mb *Spirodela polyrhiza *genome has been selected for whole genome sequencing by the DOE-JGI community-sequencing program (CSP). A reference genome within this family will be invaluable for gene discovery and evolutionary analysis of aquatic monocot species. Furthermore, from a systematic point of view, classification solely based on morphological characteristics has been a significant challenge. The most readily observed anatomical feature of the minute and highly reduced duckweeds are their fronds with or without roots. These few and somewhat variable morphological characters and rarely emerging flowers or fruits make identification of duckweeds extremely difficult even for professional taxonomists [29]. Complementing traditional classification methods with a DNA-based method would be highly applicable for such a family of species. It would permit these species to be classified in a highly reproducible and cost effective manner because DNA-based methods are independent of morphology, integrity, and developmental stage of the organism and can distinguish among species that superficially look alike [30].

Here, we present a simple and accessible protocol to barcode duckweeds and establish a sequence database against which unknown species may be compared and tentative species identifications can be validated. This database also provides a high-resolution phylogenetic resource for this important plant monocot family.

## Results

### Sampling criteria

The duckweed family consists of 38 species classified into 5 genera [21]. A worldwide collection has been characterized by genome sizes (Wang et al., ms. in prep.). From this collection, 97 ecotypes were sampled for the current work representing all five genera and 31 species (81.6% of the known species; Additional file 1). The ecotypes selected encompass the worldwide geographical distribution of duckweeds originating from different climates and geographical regions, ranging from N60° to S42° latitude and 9 m to 1287 m in altitude (Additional file 1, Figure 1). 85 ecotypes from 19 species were used for statistical calculations and candidate barcode evaluations. An additional 12 single-ecotype species were examined to determine the broader applicability of the barcode markers for identification.

**Figure 1:**
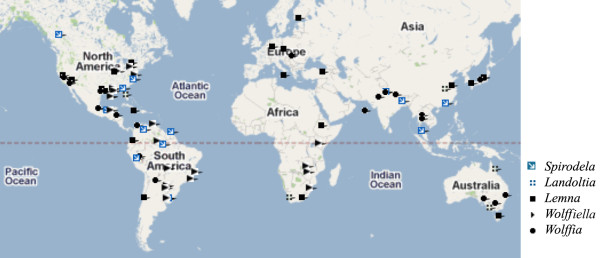
**Google map of the worldwide collection of duckweeds for the current study**. The distribution of duckweeds was made by GPS with corresponding latitude and longitude.

### Validation of DNA barcoding markers

To simplify identification of different species by DNA barcodes, a target DNA sequence marker has to meet two basic requirements: the first is a high success rate during PCR amplification and DNA sequencing, the second is sufficient DNA sequence polymorphism to permit different species to be distinguished and evolutionary distances between them to be calculated [1]. The CBOL plant-working group proposed 7 leading candidates [7], i.e., 4 coding genes (*rpoB, rpoC1, rbcL *and *matK*) and 3 noncoding spacers (*atpF-atpH, psbK-psbI *and *trnH-psbA*). To evaluate the seven markers, genomic DNA extracted from the 97 ecotypes was subjected to PCR amplification with the primer pairs based on the chloroplast sequence of *Lemna minor*. The PCR primers were also used for sequencing (See Materials and methods). PCR and sequencing were generally successful (≥95%) for all the barcode candidates except *matK *(71%) (Table 1). The maximal and minimal alignment length of PCR product for *rpoB*, *rpoC1*, *rbcL *and *matK *were identical, while that of *atpF-atpH*, *psbK-psbI *and *trnH-psbA *were quite variable, with a range of 579-622 bp, 185-576 bp and 286-504 bp, respectively. It was not unexpected that the coding markers (*rpoB*, *rpoC1*, *rbcL *and *matK*) were conserved in PCR product length, while the noncoding spacers (*atpF-atpH, psbK-psbI *and *trnH-psbA*) displayed more variability due to extensive insertions/deletions (Table 1). These results indicate that the selection of markers by the COBL plant-working group should provide a reasonable level of success for new untested plant families.

**Table 1:** Success ratios of PCR amplification and sequencing for seven candidate barcoding markers.

	*psbK*-*psbI*	*trnH*-*psbA*	*matK*	*atpF*-*atpH*	*rpoB*	*rpoC1*	*rbcL*
Max. length of product*	576	504	725	622	389	450	522
Min. length of product*	185	286	719	579	389	450	522
# tested Samples	97	97	97	97	97	97	97
% Success of PCR and sequencing	100%	95%	71%	99%	98%	100%	100%

* The analyzed product length becomes shorter than corresponding one's due to removal of the end of ambiguous nucleotides

### Intra- and inter-specific DNA sequence polymorphism

To assess the degree of DNA polymorphism between DNA samples, sequence divergences between and within species were calculated by Kimura 2-parameter (K2P) and uncorrected p-distance, respectively. Both models exhibited the same tendency: higher average interspecific diversity and lower intraspecific distance. For example, the K2P distance within and between species is as follows: *psbK*-*psbI *(0.1648 and 0.0072), *trnH*-*psbA *(0.1133 and 0.0058), *matK *(0.0715 and 0.0019), *atpF-atpH *(0.0633 and 0.0008) *rpoB *(0.0388 and 0.0069)*, rpoC1 *(0.0303 and 0.0006)*, rbcL *(0.0216 and 0.0004). The noncoding spacer *psbK*-*psbI *showed the highest interspecific diversity (66 average substitution sites among 675 bp), while the coding marker *rbcL *is the most conserved one (11 average substitution sites among 522 bp) (Table 2). Wilcoxon signed rank tests further showed that the most variable barcode between species was *psbK*-*psbI*, followed by *trnH*-*psbA*, *matK *and *atpF*-*atpH *(Additional file 2). The lowest intraspecific distance was provided by *atpF*-*atpH *and *rbcL*, whereas the highest is *trnH*-*psbA*, *psbK*-*psbI *and *matK *(Additional file 3). Although none of the seven proposed markers possessed both the highest variation between species and the lowest distance within a species, *atpF*-*atpH *seemed to show sufficient interspecific but relatively low intraspecific divergence, compared to the other six markers (Table 2, Additional file 2 and 3).

**Table 2:** Measurement of inter- and intra-specific divergences for seven barcoding markers.

Region	*psbK*-*psbI*	*trnH*-*psbA*	*matK*	*atpF*-*atpH*	*rpoB*	*rpoC1*	*rbcL*
Aligned length (bp)*	675	520	725	674	389	450	522
Mean interspecific No. of substitution	66	32	48	44	13	13	11
Mean interspecific Kimura 2-parameter distances	0.1648 ± 0.0221	0.1133 ± 0.0120	0.0715 ± 0.0061	0.0633 ± 0.0068	0.0338 ± 0.0051	0.0303 ± 0.0050	0.0216 ± 0.0038
Mean interspecific Kimura 2-parameter distances	0.0072 ± 0.0015	0.0058 ± 0.0014	0.0019 ± 0.0003	0.0008 ± 0.0002	0.0069 ± 0.0008	0.0006 ± 0.0002	0.0004 ± 0.0002
Mean interspecific P-distances	0.1435 ± 0.0156	0.0986 ± 0.0095	0.0671 ± 0.0052	0.0601 ± 0.0059	0.0327 ± 0.0048	0.0295 ± 0.0048	0.0212 ± 0.0037
Mean interspecific P-distances	0.0066 ± 0.0012	0.0057 ± 0.0014	0.0019 ± 0.0003	0.0008 ± 0.0002	0.0062 ± 0.0007	0.0006 ± 0.0002	0.0004 ± 0.0002

* Aligned length becomes longer than corresponding ones due to addition of the gap.

**Figure 2:**
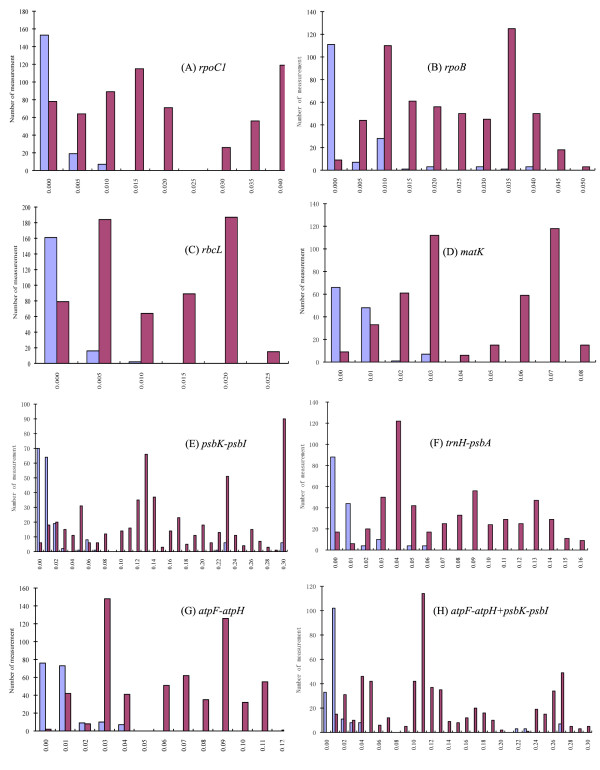
**Relative distribution of all intra- and inter-specific divergence for single or combined markers**. (A) *rpoC1*. (B) *rpoB*. (C) *rbcL*. (D) *matK*. (E) *psbK*-*psbI*. (F) *trnH*-*psbA*. (G) *atpF*-*atpH*. (H) *atpF*-*atpH*+ *psbK*-*psbI*. × axis is uncorrected p-distance with corresponding increment unit based on variation of each marker. Y axis is the number of occurrences. Barcoding gaps were evaluated with high significance (p < 0.0001) by Median and Mann-Whitney U tests for all markers. Blue bars indicate intraspecific distance and red bars are interspecific distance.

The accuracy of barcoding for species identification depended to a large extent on the barcoding gap between intraspecific and interspecific sequence variations. Effective barcoding became weaker when interspecific and intraspecific distances overlapped. To evaluate whether there was a significant barcoding gap, we calculated the distribution of divergences for the seven markers (Figure 2). Median and Mann-Whitney U tests inferred that the mean of intraspecific divergence was significantly lower than that of interspecific distance in each case (p < 0.0001). Even though *psbK*-*psbI *and *trnH*-*psbA *exhibited the highest rates of divergence between species, they were also most diverged within species, which could easily result in misidentification (Table 2, Additional file 3 and 4, Figure 2). On the other hand, the adequate variation and the narrow overlapping distance of the *atpF*-*atpH *marker would ensure accurate ecotype and species identification (Table 2, Additional file 2 and 3, Figure 2).

### DNA sequence similarity-based identification

**Figure 3:**
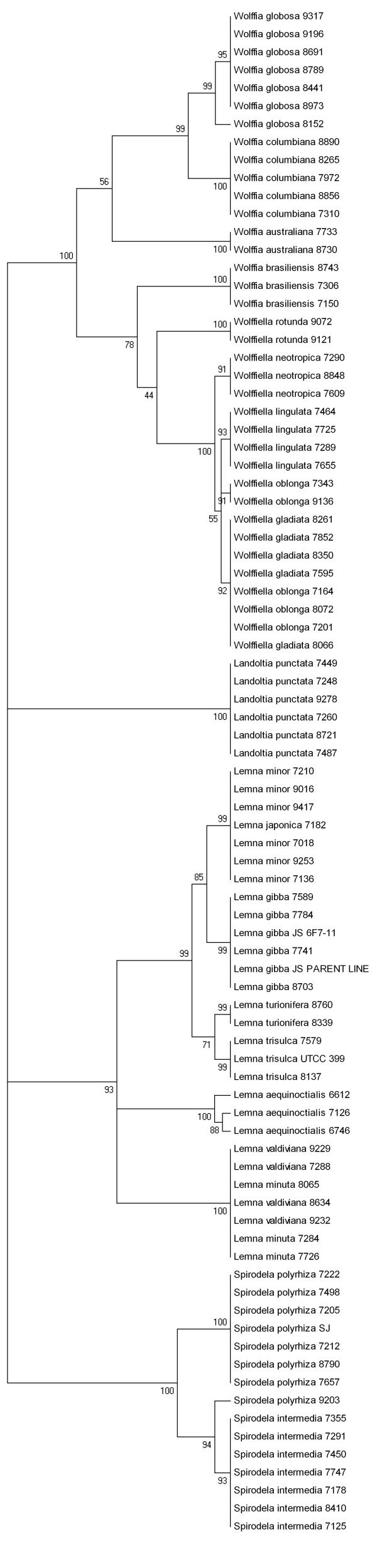
**UPGMA tree based *atpF*-*atpH *sequences**. The tree was drawn among 20 species with more than one ecotype except *L. japonica*.

In order to test whether accurate species identification can be made in our samples, we adopted the "best match" function in the program TAXONDNA [31]. The rank order for the correct identification is *atpF-atpH *(92.85%) *psbK*-*psbI *(84.7%), *trnH*-*psbA *(82.5%), *matK *(77.77%), *rpoB *(77.5%)*, rpoC1 *(70.58%)*, rbcL *(70.58%) (Table 3). Generally, the three noncoding spacers produced higher rates of successful identifications than those of the four coding markers. Consistent with Figure 3, *atpF*-*atpH *yielded the best result with 92.85% successful identifications. Among 84 ecotypes (not including species with single sampled ecotypes), 78 samples were successfully discriminated, three were ambiguous and three were incorrectly identified using *atpF*-*atpH*. When we combined *atpF*-*atpH *with one of the other five barcoding markers, the percentage of correct identification dropped, except for *psbK*-*psbI*, which gave an increase of 1.19% (Table 3). The markers *matK *+ *atpF*-*atpH *were not counted because of the small number of sequence comparisons done with *matK*.

**Table 3:** Identification success based on "best close match" tools.

	*psbK-psbl*	*trnH-psbA*	*matK*	*atp-atpH*	*rpoB*	*rpoCl*	*rbcL*	*psbK-psbl + atp F-atpH*	*trnH-psbA + atp F-atpH*	*matK+ atpF-atpH*	*rpoB+atp F-atpH*	*rpoCl + atp F-atp H*	*rbcL + atpF-atpH*
Correct	72(84.7%)	66(82.5%)	49(77.77%)	78(92.85%)	62(77.5%)	60(70.58%)	60(70.58%)	79(94.04%)	71(89.87%)	/	77(91.66%)	77(91.66%)	77(91.66%)
Ambiguous	8(9.41%)	11(13.75%)	10(15.87%)	3(3.57%)	12(15.0%)	21(24.7%)	21(24.7%)	0(0.0%)	3(3.79%)	/	2(2.38%)	4(4.76%)	4(4.76%)
Incorrect	5(5.88%)	2(2.5%)	4(6.34%)	3(3.57%)	6(7.5%)	4(4.7%)	2(2.35%)	5(5.95%)	5(6.32%)	/	5(5.95%)	3(3.57%)	3(3.57%)
No match	0(0.0%)	1(1.25%)	0(0.0%)	0(0.0%)	0(0.0%)	0(0.0%)	2(2.35%)	0(0.0%)	0(0.0%)	/	0(0.0%)	0(0.0%)	0(0.0%)
Threshold	22.12%	4.01%	2.62%	2.96%	2.57%	0.44%	0.38%	22.16%	2.08%	/	2.44%	1.77%	1.67%

"best close match" was analyzed by TAXONDNA program [31] with single region or two-region combinations. The ecotypes was classified into correct, ambiguous, incorrect and no match group. The group number was shown in each well. Number in bracket indicates percentage in all barcoding ecotypes. *matK *+ *atpF*-*atpH *was not counted due to the small number of sequence comparison done for *matK*. Percentage in the bracket was calculated by dividing each item by all tested sample.

### Tree-based sequence classification

As an alternative to sequence similarity-based identification, we estimated the proportion of recovered monophyly from multiple conspecific ecotypes per species in the phylogenetic tree for each barcoding marker. Here, we need to stress that the primary purpose of the tree is not so much the evolutionary relationship, but the species identification. The *atpF*-*atpH *attained the highest score of monophyletic species (73.7%, i.e., 14 correctly identified out of 19 species; Table 4 and Figure 3). The number of successfully identified species with the other six markers was *rpoB *(11)*, rpoC1 *(11)*, rbcL *(11), *trnH*-*psbA *(10), *psbK*-*psbI *(8). The *atpF-atpH *marker did not distinguish closely-related pairs of sister species such as *W. gladiata *and *W. oblonga *and *L. minuta *and *L. valdiviana*.

**Table 4:** Number of monophyletic species recovered with the best two phylogenetic methods for six markers

Loci	UPGMA	MP
*psbK-psbI*	8 (93.3)	8 (87.5)
*trnH-psbA*	10 (87.5)	10 (85.7)
*matK*	/	/
*atpF-atpH*	14 (100)	14 (94.1)
*rpoB*	11 (83.3)	11 (68.8)
*rpoC1*	11 (85.7)	11 (68.8)
*rbcL*	11 (85.7)	12 (68.8)

The number of monophyletic species out19 species was shown in each well. Proportions supported by bootstrap >50% are in brackets.

Although the location of most grouped ecotypes in the taxonomic trees did not change in regard to each marker, a close examination consistently revealed two interesting connections. First, despite the fact that very little is known about how cross pollination in these tiny flowering plants occurs, *L. japonica *has been suspected to originate from a hybridization event between *L. minor *and *L. turionifera *based on morphological characters [22]. Our data indicates that sequence from each of the seven tested markers of *L. japonica *7182 was always identical to and clustered with *L. minor *(Figure 3). Since the chloroplast is maternally inherited in many (but not all) plants, our data is consistent with *L. japonica *arising from a cross between *L. minor *and *L. turionifera*.

The second connection was *S. polyrhiza *9203, which consistently clusters with *S. intermedia *rather than other *S. polyrhiza *in all seven tested markers (Figure 3). We examined 34 ecotypes of *S. polyrhiza *from the collection using the *atpF*-*atpH *marker and found four additional ecotypes that grouped closely with *S. intermedia *(Additional file 4). This suggested that these accessions might have been misidentified as *S. polyrhiza *due to the overlap in morphological characteristics between these species.

## Discussion

Here, we present data validating the most useful DNA barcoding markers for the family of *Lemnaceae *from among those proposed by the CBOL plant-working group. Such a fundamental, whole family-wide analysis lays the groundwork for phylogenetic and genomic studies. Our samples represent a worldwide collection from the same family with many sister species (Figure 1 and 3, Additional file 1). Specimens in previous taxonomic classifications using barcoding markers were mainly from distantly related groups from broadly different families that originated from the local or more defined regions, such as the National Park [8], the Amazon [32], and the Panama region [14]. Because of the diversity of the collection that has accumulated over the years, duckweeds provide a unique system to test the proposed barcoding markers for closely related species. Furthermore, it is difficult to classify members of this family by morphology alone. Therefore, we can not only validate the universal application of barcoding markers, but also apply it to species that may be solely dependent on such an approach for conservation. The advantage of universal barcoding markers is the design of universal primers for barcoding markers from reference sequences, which in this case was *L. minor *[33]. The primers worked very well for all the samples (31 species and 97 ecotypes) with PCR amplification and the sequencing success rates better than 95%, except in the case of *matK*, which yielded a rate as low as 71% (Table 1). In addition, a lower PCR annealing temperature than optimal for *Lemna minor *permits primers to anneal to the target sequences despite sequence polymorphism in related species. It is interesting that most PCR failure existed in the *Wolffioideae *subfamily (Additional file 1). The locus *matK *has been shown to be very variable in numerous phylogenetic studies [34,35]} and other studies have also noted the difficulties of its utilization due to PCR failure and lack of truly universal primer sites [9,10]. Further improvement of primer designs for matK for other targets could increase amplification success, but might fail because of less conserved sites near the most variable sequences of the locus. Although *matK *DNA sequences exhibited the highest interspecific variation among the four coding markers (Table 2), the low percentage of successful PCR amplification and sequencing in duckweeds would restrict its extensive use.

It was not surprising that the noncoding spacers showed dramatically higher sequence variability than the coding markers (Table 2). Given the slow evolutionary rate of *rpoB*, *rpoC1 *and *rbcL *(especially for *rbcL*, which is strongly recommended for barcoding across all land plants), they work well to distinguish distantly related species either alone or when combined with other more variable regions [6,9]. However, their sequence polymorphisms might not be sufficient to distinguish closely related species. The non-coding spacers of *psbK*-*psbI *and *trnH*-*psbA *were the most polymorphic plastid sequences with variable sequence length in duckweeds (Table 1). The size of *trnH*-*psbA *in *Spirodela *(~504 bp) was 218 bp longer than in the other four genera (~286 bp). The length of the *psbK*-*psbI *sequence was the most variable, ranging from ~185 bp in *S. polyrhiza *to ~479 bp in *S. intermedia *even though they were sister-species (Table 1 and Figure 3). These significant length variations caused by deletion/insertion, simple sequence repeats and rearrangements were problematic for accurate alignment, but could potentially be adapted for simple diagnostic tests that would not require DNA sequencing. Furthermore, the high sequence polymorphisms of the aligned sequences of *psbK*-*psbI *and *trnH*-*psbA *could offer greater distinction between species in a diverse set of genera in certain families [5,8]. Still, one has to use caution for intraspecies comparison where the relatively higher intraspecific distance compromised their power in barcoding duckweed species. One nearly has to cluster samples into two groups, one for ecotypes of the same species and one for species to species comparison (Table 2, 3, and 4, Figure 3). Failure to do so would prevent the detection of true differences between congeneric species and conspecific ecotypes and therefore impede the use of a universal duckweed barcode (Figure 2).

Although previous studies showed that *atpF*-*atpH *as a barcoding marker was inferior to *psbK*-*psbI*, *trnH*-*psbA *and *matK *based on distantly related species [5,8,9], our data suggested that it was the most promising barcoding marker for duckweeds with respect to high PCR amplification, ease of alignment, and sufficient sequence divergence (Table 1, 2, 3, 4 and Figure 2). Therefore, our data differed from the conclusions of evaluating barcoding markers made from unrelated species. Although it was shown that barcoding plants by more than one region tended to be more effective [11-13], combination of *atpF-atpH *with any of the other markers resulted in only slight increases or drops of the rate of successful identification of species compared to itself alone (Table 3), indicating that the discriminatory power of *atpF*-*atpH *has already reached an optimum. When the *atpF*-*atpH *marker was combined with other markers, the reduced resolution lowered the differential value without complementary benefits. A similar finding that a combination of *matK *and *trnH*-*psbA *did not improve species identification has been reported as well [8].

One of the most significant applications of DNA barcoding is to overcome taxonomic obstacles, where it is difficult to identify unknown or wrongly named species in a family with similar morphology (Figure 3). Furthermore, DNA barcoding could offer us a primary screen for further characterization of cryptic species. Although scientists within the duckweed community were trying to resolve the question of whether *L. japonica *(Lj) originated from hybridization of *L. minor *(Lm) and *L. turionifera *(Lt), preliminary attempts to cross Lm and Lt (50 crosses) to reproduce the hybridization event were not successful [22]. The key problem is that flowering is very rare and the flower is small in size, which makes outcrossing extremely tedious [23]. Here, the sequences from the seven tested chloroplast markers of *L. japonica *7182 were always identical and clustered with *L. minor *(Figure 3). Therefore, we used the limited nuclear markers (glyceraldehyde-3-phosphate dehydrogenase, histone 3 gene, beta-1,2-xylosyltransferase isoform 1, expression control elements from the *Lemnaceae *family) to uncover the relationship among them by polymorphisms. Unexpectedly, the sequences showed great conservation and there was not sufficient variation to answer this question. However, the identical alleles in *L. japonica *7182 and *L. minor *support the assumption that *L. japonica *might have come from the cross of *L. minor *and *L. turionifera*.

Generally speaking of members of the duckweed family, the more derived they are, the simpler their morphologies. The reduction in size and simplification in structure make the fronds more mobile and better successfully adapt to variable conditions [22]. *S. intermedia *was characterized by a slight degree of primitivism of more nerves, roots, and ovules compared to *S. polyrhiza*, which suggested that *S. intermedia *was differentiated into *S. polyrhiza *potentially through gradual morphological reduction and isolation. However, gradual differences were sometimes difficult to distinguish from each other due to overlapping characteristics [22]. Our studies for 34 ecotypes of *S. polyrhiza *using *atpF-atpH *markers showed five ecotypes that have been clustered with *S. intermedia *(Additional file 4), which is mainly restricted to South America [22]. Good trace evidence comes from *S. polyrhiza *9203 (Figure 3). Among five ecotypes, three are derived from South America, while another two are from India. Therefore, a refined classification is necessary to determine whether another four ecotypes except *S. polyrhiza *9203 should be classified as *S. intermedia *rather than *S. polyrhiza*.

Both phylogenetic data [21] and our barcoding data showed that closely related species *W. gladiata *and *W. oblonga*, *L. minuta *and *L. valdiviana *could not be separated from each other (Figure 3). These sister-species share identical sequences for barcoding markers, which would require a search for additional barcoding markers with greater sequence polymorphism. In fact, a universal DNA barcoding marker has not been reported to distinguish more than 90% of species tested until now [8,32]. Elucidation of recently evolved species sharing identical barcoding sequences still needs further taxonomic or case-by-case morphological, flavonoid, and allozyme analyses. On the other hand, use of next-generation sequencing technologies and corresponding software applications are emerging where low pass coverage of different specimen could provide the necessary resolution.

## Conclusions

In this study we have demonstrated that *atpF-atpH *noncoding spacer could serve as a universal DNA barcoding marker for species-level identification of duckweeds. This marker will allow to identify unknown species or to exploit new species of duckweeds by reason of its reliable amplification, straightforward sequence alignment, and rates of DNA variation between species and within species. DNA barcoding developed in this study are a significant contribution to the taxonomical structure in duckweeds compared with insensitive morphological classification.

## Methods

### Plant materials

The *Lemnaceae *collection originated from the Institut für Integrative Biologie (Zürich, Switzerland), the BIOLEX company (North Carolina, USA), and the University of Toronto Culture Collection of Algae and Cyanobacteria (UTCC, Toronto, Canada) where it was maintained for many years. Detailed information about many of these accessions is included in Dr. Landolt's monographic study [29]. In total, 97 ecotypes representing 31 species (81.6% of the known species) were sampled in this study. Since the intraspecific distance is very important for evaluating a suitable barcoding marker, 2 to 8 representatives per species are included for 19 species, whereas another 12 species are represented by a single ecotype. Moreover, the selected ecotypes represent a worldwide geographical distribution (Figure 1). A summary of all specimens included in this study was listed in Additional file 1.

### DNA amplification, sequencing and alignment

All duckweed fronds were grown aseptically in half-strength Schenk and Hildebrandt medium (Sigma, S6765). Total DNA was extracted using CTAB [36]. The chloroplast markers *rpoB, rpoC1, rbcL, matK*, *atpF-atpH, trnH-psbA*, and *psbK-psbI*, which were proposed by the CBOL plant-working group, were amplified with a set of modified primers (Table 5) based on reference sequences from *Lemna minor *[33]. The amplicon sizes were also estimated according to *Lemna minor *(Table 5). PCR reaction conditions also followed guidelines from the CBOL plant-working group. Briefly, 50-100 ng genomic DNA and 5 pmol of each primer are added with the JumpStart™Redtop^® ^ReadyMix™Reaction Mix (P1107, Sigma) Redix in 25 ml of final volume. To improve the universal application of these primers, they were designed to have an annealing temperature (T_a_) of 50°C, which is 1 to 6°C lower than the optimal T_a _of *Lemna minor *as determined by Beacon Designer software (PREMIER Biosoft International) under reaction conditions of 50 mM monovalent ion and 200 nM nucleic acid concentration (Table 5). The program uses the following formula: optimal T_a _= 0.3 × Tm (primer) + 0.7 Tm (product) -14.9 [37]. The PCR products were purified with ExoSap-IT™(USB Corp.) and then sequenced on an ABI3730 automated sequencer using the same primers as in the PCR reactions. Both strands of each PCR product were sequenced and double-checked. The success ratios of PCR amplification and sequencing were counted (Table 1). After the ambiguous nucleotides (~30bp) at the ends of reads were removed, the length of products was measured and multiple DNA sequence alignments were generated using ClustalW in MEGA 4.1 [38].

**Table 5:** List of primers for the seven proposed DNA barcoding markers.

Marker	Primer sequence	Amplicon size (*Lemna minor*)	Ta Optimum (*Lemna minor*)
*psbK-psbI*	Forward: 5'-TTAGCATTTGTTTGGCAAG-3';	544 bp	51°C
	Reverse: 5'- AAAGTTTGAGAGTAAGCAT -3'		
*trnH-psbA*	Forward: 5'-GTTATGCACGAACGTAATGCTC-3';	300 bp	55°C
	Reverse: 5'- CGCGCGTGGTGGATTCACAATCC-3'		
*matK*	Forward: 5'-CGTACTGTACTTTTATGTTTACGAG-3';	862 bp	55°C
	Reverse: 5'- ATCCGGTCCATCTAGAAATATTGGTTC -3'		
*atpF-atpH*	Forward: 5'-ACTCGCACACACTCCCTTTCC-3';	675 bp	53°C
	Reverse: 5'- GCTTTTATGGAAGCTTTAACAAT -3'		
*rpoB*	Forward: 5'-ATGCAGCGTCAAGCAGTTCC-3';	406 bp	55°C
	Reverse: 5'- TCGGATGTGAAAAGAAGTATA -3'		
*rpoC1*	Forward: 5'-GGAAAAGAGGGAAGATTCCG-3';	509 bp	56°C
	Reverse: 5'- CAATTAGCATATCTTGAGTTGG -3'		
*rbcL*	Forward: 5'-GTAAAATCAAGTCCACCACG-3';	580 bp	56°C
	Reverse: 5'-ATGTCACCACAAACAGAGACTAAAGC -3'		

### Genetic distance analysis

Genetic distance was calculated using pairwise alignments of sequences between and within species (Table 2). The average intraspecific distance was calculated with the mean pairwise distance in each species with more than one representative, which eliminated biases due to unbalanced sampling among taxa. We evaluated conspecific and congeneric variability for each pair of marker sequences by Wilcoxon signed rank tests (Additional file 2 and 3) [9]. Median and Mann-Whitney U tests were executed to examine the extent of DNA barcoding gap/overlap between intra- and inter-specific divergences [8].

### Evaluation of DNA barcoding markers based on sequence similarity

For assessing success in species assignment or identification among our data set, we adopted the "best match" function in the program TAXONDNA (Table 3) [31]. We calculated pairwise distances as uncorrected pairwise distances and compared two sequences over at least 300 bp except for *psbK-psbI *(230 bp). We suppressed indels when computing distances. The threshold was set at a value below which 95% of all intraspecific pairwise distances were found. Since the best match was based on direct sequence comparison with other conspecific ecotypes, the analysis only counted species with multiple ecotypes per species.

### Evaluation of DNA barcoding markers using phylogenetic analysis

The other criterion used to measure success of species identification was based on generating a phylogenetic tree. We built trees with MEGA 4.1 by using the best algorithms methods of UPGMA and MP compared with other tree building techniques for DNA barcoding [8]. UPGMA trees were made from K2P distances. The MP trees were constructed using the close neighbor interchange (CNI) method with search level 1. The initial tree for the CNI search was created by random addition for 10 replications. Each tree contains the bootstrap values as calculated by the software from 500 replicates. Here, we only calculated the number of successfully clustered species as monophyly among the species with multiple conspecific individuals (Figure 3, Additional file 4, Table 4).

## Authors' contributions

WW designed experiment, analyzed data and wrote the manuscript. YW conducted PCR and sequenced the products. YY and ME kept the duckweeds collection and extracted genomic DNA. RK provided advices for experiments and revised manuscript. JM supervised the work, interpreted data with WW, and revised all versions of the manuscript. All authors read and approved the final manuscript.

## Supplementary Material

Additional file 1 **Information of sampled duckweeds and GenBank accession numbers for sequence**. A complete list of all species and ecotypes with relevant information including geographical position and marker sequences is provided. Click here for file

Additional file 2 **Wilcoxon signed rank tests of interspecific distance among markers**. Values for each marker assessment is provided and ordered. Click here for file

Additional file 3 **Wilcoxon signed rank tests of intraspecific divergence among markers**. Values for each marker assessment is provided and ordered. Click here for file

Additional file 4 **UPGMA tree based atpF-atpH sequences for sister species of S. polyrhiza and S. intermedia**. Distance analysis was carried out as described under Methods. Click here for file
