# Supplementary material for: DNA barcoding of the Lemnaceae, a family of aquatic monocots
Source: BMC Plant Biol. 2010 Sep 16;10:205. doi: 10.1186/1471-2229-10-205 (PMC2956554; doi:10.1186/1471-2229-10-205)
Supplement: Additional file 1 — Information of sampled duckweeds and GenBank accession numbers for sequence. A complete list of all species and ecotypes with relevant information including geographical position and marker sequences is provided. [file 1471-2229-10-205-S1.PDF]

Species name Checked on IPNI	Landolt#	Location	GPS	Altitude (m)	*psbK-psbI*	*trnH-psbA*	*matK*	*atpF-atpH*	*rpoB*	*rpoC1*	*rbcL*
Spirodela intermedia	7125	Uruguay,Canelones, Carrasco	S34º W56º	547	GU454290		GU454125	GU454194	GU454030	GU453933	GU454387
Spirodela intermedia	7178	Argentina,Buenos Aires, Buenos Aires	S34º W58º	548	GU454291		GU454126	GU454195	GU454031	GU453934	GU454388
Spirodela intermedia	7291	Brazil,Amazonas, Manaus, Parana Channel	S3º W60º	550	GU454292	GU454484	GU454127	GU454196	GU454032	GU453935	GU454389
Spirodela intermedia	7355	Surinam,Paramaribo, Charlesburg	N5º W55º	567	GU454293	GU454485	GU454128	GU454197	GU454033	GU453936	GU454390
Spirodela intermedia	7450	India,Delhi, Botanical Garden	N28º E77º	555	GU454294		GU454129	GU454198	GU454034	GU453937	GU454391
Spirodela intermedia	7747	Peru,Lima, San Marcos Bot. Garden	S11º W76º	558	GU454295	GU454486	GU454130	GU454199	GU454035	GU453938	GU454392
Spirodela intermedia	8410	Panama,Panama City	N30º W85º	563	GU454296		GU454131	GU454200	GU454036	GU453939	GU454393
Spirodela polyrhiza	7205	Hong Kong,New Territories, Shatin	N22º E114º	643	GU454297	GU454487	GU454132	GU454201	GU454037	GU453940	GU454394
Spirodela polyrhiza	7212	India,West Bengal, Rajnagar	N23º E87º	1253	GU454298	GU454488	GU454133	GU454202	GU454038	GU453941	GU454395
Spirodela polyrhiza	7222	Malaysia,Selangor, Kuala Lumpur	N3º E101º	646	GU454299	GU454489	GU454134	GU454203	GU454039	GU453942	GU454396
Spirodela polyrhiza	7498	USA,North Carolina, Durham Co., Durham	N35º W78º	658	GU454300	GU454490	GU454135	GU454204	GU454040	GU453943	GU454397
Spirodela polyrhiza	7657	Mexico,Veracruz, Coatzacoalcos	N18º W94º	663	GU454301	GU454491	GU454136	GU454205	GU454041	GU453944	GU454398
Spirodela polyrhiza	8790	Canada,British Columbia, Vanderhoof	N54º W124º	702	GU454302	GU454492	GU454137	GU454206	GU454042	GU453945	GU454399
Spirodela polyrhiza	9203	Colombia,Huila, El Juncal	N6º W72º	1202	GU454303		GU454138	GU454207	GU454043	GU453946	GU454400
Spirodela polyrhiza	SJ	Europe			GU454304	GU454493	GU454139	GU454208	GU454044	GU453947	GU454401
Landoltia punctata	7248	South Africa,Cape, Stellanbosch, Jonkershoek	S33º E18º	589	GU454305	GU454494	GU454140	GU454209	GU454045	GU453948	GU454402
Landoltia punctata	7260	Victoria,Tyrendarra	S38º E141º	590	GU454306	GU454495	GU454141	GU454210	GU454046	GU453949	GU454403
Landoltia punctata	7449	India,Delhi	N28º E77º	597	GU454307	GU454496	GU454142	GU454211	GU454047	GU453950	GU454404
Landoltia punctata	7487	USA,Florida, Polk Co., Plant City	N27º W82º	600	GU454308	GU454497	GU454143	GU454212	GU454048	GU453951	GU454405
Landoltia punctata	8721	Queensland,Atherton, the Crater	S17º E145º	616	GU454309	GU454498	GU454144	GU454213	GU454049	GU453952	GU454406
Landoltia punctata	9278	China,Wuhan University	N30º E114º		GU454310	GU454499	GU454145	GU454214	GU454050	GU453953	GU454407
Lemna aequinoctialis	6612	USA,California, Fresno Co., Centerville	N36º W120º	131	GU454311	GU454500	GU454146	GU454215	GU454051	GU453954	GU454408
Lemna aequinoctialis	6746	USA,California, Merced Co., Plainsburg	N37º W121º	132	GU454312	GU454501	GU454147	GU454216	GU454052	GU453955	GU454409
Lemna aequinoctialis	7126	USA,Texas, Travis Co., Austin (university pond)	N30º W97º	136	GU454313	GU454502	GU454148	GU454217	GU454053	GU453956	GU454410
Lemna disperma	7269	Tasmania,Sorell	S42º E147º	273	GU454314	GU454503	GU454149	GU454218	GU454054	GU453957	GU454411
Lemna gibba	7589	USA,California, Los Angeles Co., Covina	N34º W117º	317	GU454315	GU454504	GU454150	GU454219	GU454055	GU453958	GU454412
Lemna gibba	7741	Italy,Sicilia, Siracusa (G3)	N37º E15º	324	GU454316	GU454505	GU454151	GU454220	GU454056	GU453959	GU454413
Lemna gibba	7784	Ethiopia,Shoa, 30 km E of Addis Abeba	N9º E38º	327	GU454317	GU454506	GU454152	GU454221	GU454057	GU453960	GU454414
Lemna gibba	8703	Japan,Honshu Aichi	N36º E138º	344	GU454318	GU454507	GU454153	GU454222	GU454058	GU453961	GU454415
Lemna gibba	JS 6F7-11				GU454319	GU454508	GU454154	GU454223	GU454059	GU453962	GU454416
Lemna gibba	JS parent line				GU454320	GU454509	GU454155	GU454224	GU454060	GU453963	GU454417
Lemna japonica	7182	Japan,Kyushu, Fukuoka, Mizumaki-machi	N33º E130º	357	GU454321	GU454510	GU454156	GU454225	GU454061	GU453964	GU454418

**Species name Checked on IPNI**	**Landolt#**	**Location**	**GPS**	**Altitude (m)**	***psbK-psbI***	***trnH-psbA***	***matK***	***atpF-atpH***	***rpoB***	***rpoC1***	***rbcL***
Lemna minor	7018	Turkey,Anatatolia, Erzincan	N39º E39º	9	GU454322	GU454511	GU454157	GU454226	GU454062	GU453965	GU454419
Lemna minor	7136	USA,Illinois, Know Co., Williamstead	N39º W89º	15	GU454323	GU454512	GU454158	GU454227	GU454063	GU453966	GU454420
Lemna minor	7210	South Africa,Cape, Grahamtown, "Rockeby Park"	S33º E26º	21	GU454324	GU454513	GU454159	GU454228	GU454064	GU453967	GU454421
Lemna minor	9016	Japan,Honshu, Hyogo, Akashi	N36º E138º	70	GU454325	GU454514	GU454160	GU454229	GU454065	GU453968	GU454422
Lemna minor	9253	Finland,Uusimaa, Espoo	N60º E24º	1242	GU454326	GU454515	GU454161	GU454230	GU454066	GU453969	GU454423
Lemna minor	9417	Germany,Marburg (original strain of Pirson 1950)	N50º E8º		GU454327	GU454516	GU454162	GU454231	GU454067	GU453970	GU454424
Lemna minuta	7284	Uruguay,Montevideo, Canelones, Carrasco	S34º W56º	1219	GU454328	GU454517	GU454163	GU454232	GU454068	GU453971	GU454425
Lemna minuta	7726	Chile,Valparaiso, Limache	S33º W71º	97	GU454329	GU454518	GU454164	GU454233	GU454069	GU453972	GU454426
Lemna minuta	8065	USA,Texas, Brazoria Co., Old Ocean	N29º W95º	99	GU454330	GU454519	GU454165	GU454234	GU454070	GU453973	GU454427
Lemna obscura	7856	USA,Louisiana, East Baton Rouge Par., L.S.U.	N30º W91º	1177	GU454331	GU454520	GU454166	GU454235	GU454071	GU453974	GU454428
Lemna trisulca	7579	Canada,Ontario, York Co., Ajax	N43º W79º	429	GU454332	GU454521	GU454167	GU454236	GU454072	GU453975	GU454429
Lemna trisulca	8137	USA,California, San Bernardino Co., Big Bear L.	N35º W115º	440	GU454333	GU454522	GU454168	GU454237	GU454073	GU453976	GU454430
Lemna trisulca	UTCC 399				GU454334	GU454523	GU454169	GU454238	GU454074	GU453977	GU454431
Lemna turionifera	8339	China,Kiangsu, Nanking, Lake Sans-souci	N32º E118º	362	GU454335	GU454524	GU454170	GU454239	GU454075	GU453978	GU454432
Lemna turionifera	8760	Czech.,Natolice	N49º E15º	1239	GU454336	GU454525	GU454171	GU454240	GU454076	GU453979	GU454433
Lemna valdiviana	7288	Brazil,Amazonas, Manaus, Rio Negro	S1º W63º	500	GU454337	GU454526	GU454172	GU454241	GU454077	GU453980	GU454434
Lemna valdiviana	8634	Jamaica,Manchester Par., Hole River	N18º W77º	106	GU454338	GU454527	GU454173	GU454242	GU454078	GU453981	GU454435
Lemna valdiviana	9229	Ecuador,Pichincha, Rio Chiche	S0º W78º	1257	GU454339	GU454528	GU454174	GU454243	GU454079	GU453982	GU454436
Lemna valdiviana	9232	Ecuador,Pichincha, between Tandapi and Aloag	S0º W78º	1258	GU454340	GU454529	GU454175	GU454244	GU454080	GU453983	GU454437
Wolffiella denticulata	8221	South Africa,Natal, Sordwana Bay	S28º E30º	984	GU454341	GU454530	GU454176	GU454245	GU454081	GU453984	GU454438
Wolffiella gladiata	7595	USA,Virginia, Prince George Co., Brandon	N37º W77º	990	GU454342	GU454531	GU454177	GU454246	GU454082	GU453985	GU454439
Wolffiella gladiata	7852	USA,Louisana, East Baton Rouge Par., 15 km S of LSU	N30º W91º	991	GU454343	GU454532	GU454178	GU454247	GU454083	GU453986	GU454440
Wolffiella gladiata	8066	USA,Texas, Matagorda Co., Old Ocean to Cedar Lane	N28º W96º	992	GU454344	GU454533	GU454179	GU454248	GU454084	GU453987	GU454441
Wolffiella gladiata	8261	USA,Pennsylvania, Crawford Co., Conneaut L.	N41º W80º	993	GU454345	GU454534		GU454249	GU454085	GU453988	GU454442
Wolffiella gladiata	8350	USA,Illinois, Union Co, Pine Hills Swamps	N39º W88º	994	GU454346	GU454535		GU454250	GU454086	GU453989	GU454443
Wolffiella hyalina	8640	Tanzania,Arusha, Amboseli	S2º E36º	1003	GU454347	GU454536	GU454180	GU454251	GU454087	GU453990	GU454444
Wolffiella lingulata	7289	Brazil,Amazonas, Manaus, Rio Negro	S1º W63º	1007	GU454348	GU454537		GU454252	GU454088	GU453991	GU454445
Wolffiella lingulata	7464	Venezuela,Yaracuy, Marin	N10º W66º	1011	GU454349	GU454538	GU454181	GU454253	GU454089	GU453992	GU454446
Wolffiella lingulata	7655	Mexico,Tabasco, Villahermosa	N18º W92º	1013	GU454350	GU454539	GU454182	GU454254		GU453993	GU454447
Wolffiella lingulata	7725	Argentina,Corrientes, Mburucuya, "Santa Teresa"	S28º W58º	1015	GU454351	GU454540	GU454183	GU454255	GU454090	GU453994	GU454448
Wolffiella lingulata	8742	Argentina,Corrientes, San Cosme	S27º W58º	1023	GU454352	GU454541			GU454091	GU453995	GU454449
Wolffiella neotropica	7290	Brazil,Amazonas, Neptunia, Rio Negro	S1º W63º	1056	GU454353	GU454542	GU454184	GU454256	GU454092	GU453996	GU454450
Wolffiella neotropica	7609	Brazil,Espirito-Santo, Heliofila	S19º W40º	1057	GU454354	GU454543	GU454185	GU454257	GU454093	GU453997	GU454451

Species name Checked on IPNI	Landolt#	Location	GPS	Altitude (m)	*psbK-psbI*	*trnH-psbA*	*matK*	*atpF-atpH*	*rpoB*	*rpoC1*	*rbcL*
Wolffiella neotropica	8848	Brazil,Rio de Janeiro, Barra do Marico	S23º W43º	1058	GU454355	GU454544	GU454186	GU454258	GU454094	GU453998	GU454452
Wolffiella oblonga	7164	USA,Louisiana, Oreleans Par., New Orleans	N29º W90º	1063	GU454356	GU454545	GU454187	GU454259	GU454095	GU453999	GU454453
Wolffiella oblonga	7201	Argentina,Buenos Aires, Arroyo Burgueno	S34º W58º	1065	GU454357	GU454546		GU454260	GU454096	GU454000	GU454454
Wolffiella oblonga	7343	Argentina,Tucuman, El Capital	S34º W58º	1066	GU454358	GU454547		GU454261	GU454097	GU454001	GU454455
Wolffiella oblonga	8072	USA,Texas, Matagorda Co., Old Ocean to Cedar Lane	N28º W96º	1075	GU454359	GU454548		GU454262	GU454098	GU454002	GU454456
Wolffiella oblonga	9136	Brazil,Mato Grosso, Corumba	S17º W57º	1086	GU454360	GU454549	GU454188	GU454263	GU454099	GU454003	GU454457
Wolffiella rotunda	9072	Zimbabwe,Mana Pools Nat. Park, NW of Mana Pools	S19º E29º	1285	GU454361	GU454550	GU454189	GU454264	GU454100	GU454004	GU454458
Wolffiella rotunda	9121	Zimbabwe,Urungwe Safari Area, SE of Chirundu	S16º E28º	1216	GU454362	GU454551	GU454190	GU454265	GU454101	GU454005	GU454459
Wolffia angusta	7476	Victoria,Shepparton, Bunbartha	S36º E145º	711	GU454363	GU454552		GU454266	GU454102	GU454006	GU454460
Wolffia arrhiza	8872	Hungary,Szarvas	N46º E20º	753	GU454364	GU454553		GU454267	GU454103	GU454007	GU454461
Wolffia australiana	7733	South Australia,Mount Lofty Range, Torrens Gorge	S34º E138º	766	GU454365	GU454554	GU454191	GU454268	GU454104	GU454008	GU454462
Wolffia australiana	8730	New South Wales, Singleton, Doughboy Hollow	S32º E151º	769	GU454366	GU454555	GU454192	GU454269	GU454105	GU454009	GU454463
Wolffia borealis	9123	USA,California, San Diego Co., Lake Hodges Dam	N33º W117º	1207	GU454367	GU454556		GU454270	GU454106	GU454010	GU454464
Wolffia brasiliensis	7150	USA,Texas, Hays Co., San Marcos	N29º W98º	784	GU454368	GU454557		GU454271	GU454107	GU454011	GU454465
Wolffia brasiliensis	7306	Mexico,Mexico City, Mixquic	N19º W99º	786	GU454369	GU454558		GU454272	GU454108	GU454012	GU454466
Wolffia brasiliensis	8743	Argentina,Corrientes, San Cosme	S27º W58º	809	GU454370	GU454559		GU454273	GU454109	GU454013	GU454467
Wolffia columbiana	7310	Mexico,Mexico City, Mixquic	N19º W99º	862	GU454371	GU454560		GU454274	GU454110	GU454014	GU454468
Wolffia columbiana	7972	USA,Alabama, Limestone Co., Beaverdm Creek	N30º W87º	880	GU454372	GU454561		GU454275	GU454111	GU454015	GU454469
Wolffia columbiana	8265	USA,Pennsylvania, Crawford Co., Conneaut L.	N41º W80º	884	GU454373	GU454562		GU454276	GU454112	GU454016	GU454470
Wolffia columbiana	8856	Argentina,Salta, El Rey	S24º W64º	888	GU454374	GU454563		GU454277	GU454113	GU454017	GU454471
Wolffia columbiana	8890	Mexico,S of San Pedro Sula	N15º W88º	890	GU454375	GU454564		GU454278	GU454114	GU454018	GU454472
Wolffia cylindracea	9080	Zimbabwe,Wankie Nat. Park, Ngamo Wind Pump	S19º E29º	913	GU454376	GU454565		GU454279	GU454115	GU454019	GU454473
Wolffia elongata	9188	Colombia,Atlantico, Sabanagrande-Santo Tomas	S10º W74º	1211	GU454377	GU454566		GU454280	GU454116	GU454020	GU454474
Wolffia globosa	8152	USA,California, Fresno Co., Fresno	N36º W120º	1151	GU454378	GU454567		GU454281	GU454117	GU454021	GU454475
Wolffia globosa	8441	Thailand,Bangkok	N13º E100º	949	GU454379	GU454568		GU454282	GU454118	GU454022	GU454476
Wolffia globosa	8691	Japan,Honshu, Oska, Matsubara	N35º E136º	950	GU454380	GU454569		GU454283	GU454119	GU454023	GU454477
Wolffia globosa	8789	Nepal,Bharatpur	N27º E84º	953	GU454381	GU454570		GU454284	GU454120	GU454024	GU454478
Wolffia globosa	8973	Thailand,Prachnap Khiri Khan	N15º E100º	960	GU454382	GU454571		GU454285	GU454121	GU454025	GU454479
Wolffia globosa	9196	Colombia,Cordoba, Lorica	N9º W75º	964	GU454383	GU454572	GU454193	GU454286	GU454122	GU454026	GU454480
Wolffia globosa	9317	India,Rajasthan, Ajmer Lake	N26º E73º		GU454384	GU454573		GU454287	GU454123	GU454027	GU454481
Wolffia microscopica	9276	India,Delhi ?	N28º E77º	1287	GU454385	GU454574		GU454288	GU454124	GU454028	GU454482
Wolffia neglecta	9149	Pakistan,Karachi, Gulshan-e-Iasbah	N24º E67º	969	GU454386	GU454575		GU454289		GU454029	GU454483
