# Supplementary material for: DNA barcoding of the Lemnaceae, a family of aquatic monocots
Source: BMC Plant Biol. 2010 Sep 16;10:205. doi: 10.1186/1471-2229-10-205 (PMC2956554; doi:10.1186/1471-2229-10-205)
Supplement: Additional file 2 — Wilcoxon signed rank tests of interspecific distance among markers. Values for each marker assessment is provided and ordered. [file 1471-2229-10-205-S2.PDF]

W+	W-	Relative Ranks, *n*, *p* value	Result
psbK-psbI	*trnH-psbA*	W+ = 85351, W- = 21602, *n* = 465, *p* ≤ 6.19E-29	*psbK-psbI*>>*trnH-psbA*
psbK-psbI	*matK*	W+ = 26400, W- = 166, *n* = 231, *p* ≤ 7.51E-39	*psbK-psbI*>>*matK*
psbK-psbI	*atpF-atpH*	W+ = 107293, W- = 123, *n* = 465, *p* ≤ 1.55E-77	*psbK-psbI*>>*atpF-atpH*
psbK-psbI	*rpoB*	W+ = 94355, W- = 40, *n* = 435, *p* ≤ 5.01E-73	*psbK-psbI*>>*rpoB*
psbK-psbI	*rpoC1*	W+ = 107409, W- = 7, *n* = 465, *p* ≤ 7.3E-78	*psbK-psbI*>>*rpoC1*
psbK-psbI	*rbcL*	W+ = 107412, W- = 4, *n* = 465, p ≤ 7.15E-78	*psbK-psbI*>>*rbcL*
trnH-psbA	*matK*	W+ = 15553, W- = 11243, *n* = 231, *p* ≤ 0.017	*trnH-psbA*>>*matK*
trnH-psbA	*atpF-atpH*	W+ = 96280, W- = 10211, *n* = 465, *p* ≤ 2.11E-51	*trnH-psbA*>>*atpF-atpH*
trnH-psbA	*rpoB*	W+ = 94365, W- = 30, *n* = 435, *p* ≤ 4.68E-73	*trnH-psbA*>>*rpoB*
trnH-psbA	*rpoC1*	W+ = 106906.5, W- = 46.5, *n* = 465, *p* ≤ 1.37E-77	*trnH-psbA*>>*rpoC1*
trnH-psbA	*rbcL*	W+ = 107413, W- = 3, *n* = 465, *p* ≤ 7.1E-78	*trnH-psbA*>>*rbcL*
matK	*atpF-atpH*	W+ = 23718, W- = 3078, *n* = 231, *p* ≤ 1.66E-24	*matK*>>*atpF-atpH*
matK	*rpoB*	W+ = 26744, W- = 52, *n* = 231, *p* ≤ 1.18E-39	*matK*>>*rpoB*
matK	*rpoC1*	W+ = 26796, W- = 0, *n* = 231, *p* ≤ 5.98E-40	*matK*>>*rpoC1*
matK	*rbcL*	W+ = 26796, W- = 0, *n* = 231, *p* ≤ 5.98E-40	*matK*>>*rbcL*
atpF-atpH	*rpoB*	W+ = 91569, W- = 2393, *n* = 435, *p* ≤ 5.93E-66	*atpF*-atpH>>*rpoB*
atpF-atpH	*rpoC1*	W+ = 106246, W- = 707, *n* = 465, *p* ≤ 9.78E-76	*atpF*-atpH>>*rpoC1*
atpF-atpH	*rbcL*	W+ = 106756, W- = 197, *n* = 465, *p* ≤ 3.6E-77	*atpF*-atpH>>*rbcL*
rpoB	*rpoC1*	W+ = 77052, W- = 17344, *n* = 435, *p* ≤ 1.69E-30	*rpoB*>>*rpoC1*
rpoB	*rbcL*	W+ = 91686, W- = 1842, *n* = 435, *p* ≤ 2.3E-67	*rpoB*>>*rbcL*
rpoC1	*rbcL*	W+ = 84234, W- = 19506, *n* = 465, *p* ≤ 4.51E-31	*rpoC1*>>*rbcL*
