# Supplementary material for: DNA barcoding of the Lemnaceae, a family of aquatic monocots
Source: BMC Plant Biol. 2010 Sep 16;10:205. doi: 10.1186/1471-2229-10-205 (PMC2956554; doi:10.1186/1471-2229-10-205)
Supplement: Additional file 3 — Wilcoxon signed rank tests of intraspecific divergence among markers. Values for each marker assessment is provided and ordered. [file 1471-2229-10-205-S3.PDF]

W+	W-	Relative Ranks, *n*, *p* value	Result
trnH-psbA	*psbK-psbI*	W+ = 62, W- = 43, *n* = 20, *p* ≤ 0.583	*trnH-psbA* = *psbK-psbI*
trnH-psbA	*matK*	W+ = 78, W- = 27, *n* = 17, *p* ≤ 0.1189	*trnH-psbA* = *matK*
trnH-psbA	*rpoB*	W+ = 100, W- = 20, *n* = 20, *p* ≤ 0.0108	*trnH-psbA* > *rpoB*
trnH-psbA	*rpoC1*	W+ = 97, W- = 8, *n* = 20, *p* ≤ 0.0015	*trnH-psbA* > *rpoC1*
trnH-psbA	*atpF*-atpH	W+ = 98, W- = 7, *n* = 20, *p* ≤ 0.0012	*trnH-psbA* > *atpF-atpH*
trnH-psbA	*rbcL*	W+ = 91, W- = 0, *n* = 20, *p* ≤ 0.0001	*trnH-psbA* > *rbcL*
psbK-psbI	*matK*	W+ = 45, W- = 33, *n* = 17, *p* ≤ 0.6772	*psbK-psbI* = *matK*
psbK-psbI	*rpoB*	W+ = 58, W- = 20, *n* = 20, *p* ≤ 0.1514	*psbK-psbI* = *rpoB*
psbK-psbI	*rpoC1*	W+ = 48, W- = 18, *n* = 20, *p* ≤ 0.2601	*psbK-psbI* = *rpoC1*
psbK-psbI	*atpF-atpH*	W+ = 59, W- = 19, *n* = 20, *p* ≤ 0.1294	*psbK-psbI* = *atpF-atpH*
psbK-psbI	*rbcL*	W+ = 48, W- = 7, *n* = 20, *p* ≤ 0.0186	*psbK-psbI* > *rbcL*
matK	*rpoB*	W+ = 67, W- = 29, *n* = 17, *p* ≤ 0.4263	*matK* = *rpoB*
matK	*rpoC1*	W+ = 79, W- = 12, *n* = 17, *p* ≤ 0.0085	*matK* > *rpoC1*
matK	*atpF-atpH*	W+ = 74, W- = 4, *n* = 17, *p* ≤ 0.0017	*matK* > *atpF-atpH*
matK	*rbcL*	W+ = 77, W- = 1, *n* = 17, *p* ≤ 0.0005	*matK* > *rbcL*
rpoB	*rpoC1*	W+ = 42, W- = 36, *n* = 20, *p* ≤ 0.8501	*rpoB* = *rpoC1*
rpoB	*atpF-atpH*	W+ = 44, W- = 34, *n* = 20, *p* ≤ 0.7334	*rpoB* = *atpF-atpH*
rpoB	*rbcL*	W+ = 49, W- = 17, *n* = 20, *p* ≤ 0.1748	*rpoB* = *rbcL*
rpoC1	*atpF-atpH*	W+ = 45, W- = 33, *n* = 20, *p* ≤ 0.6772	*rpoC1* = *atpF-atpH*
rpoC1	*rbcL*	W+ = 30, W- = 6, *n* = 20, *p* ≤ 0.1094	*rpoC1* = *rbcL*
atpF-atpH	*rbcL*	W+ = 44, W- = 11, *n* = 20, *p* ≤ 0.1055	*atpF-atpH* = *rbcL*
