# Supplementary figures and images for: DNA barcoding of the Lemnaceae, a family of aquatic monocots
Source: BMC Plant Biol. 2010 Sep 16;10:205. doi: 10.1186/1471-2229-10-205 (PMC2956554; doi:10.1186/1471-2229-10-205)

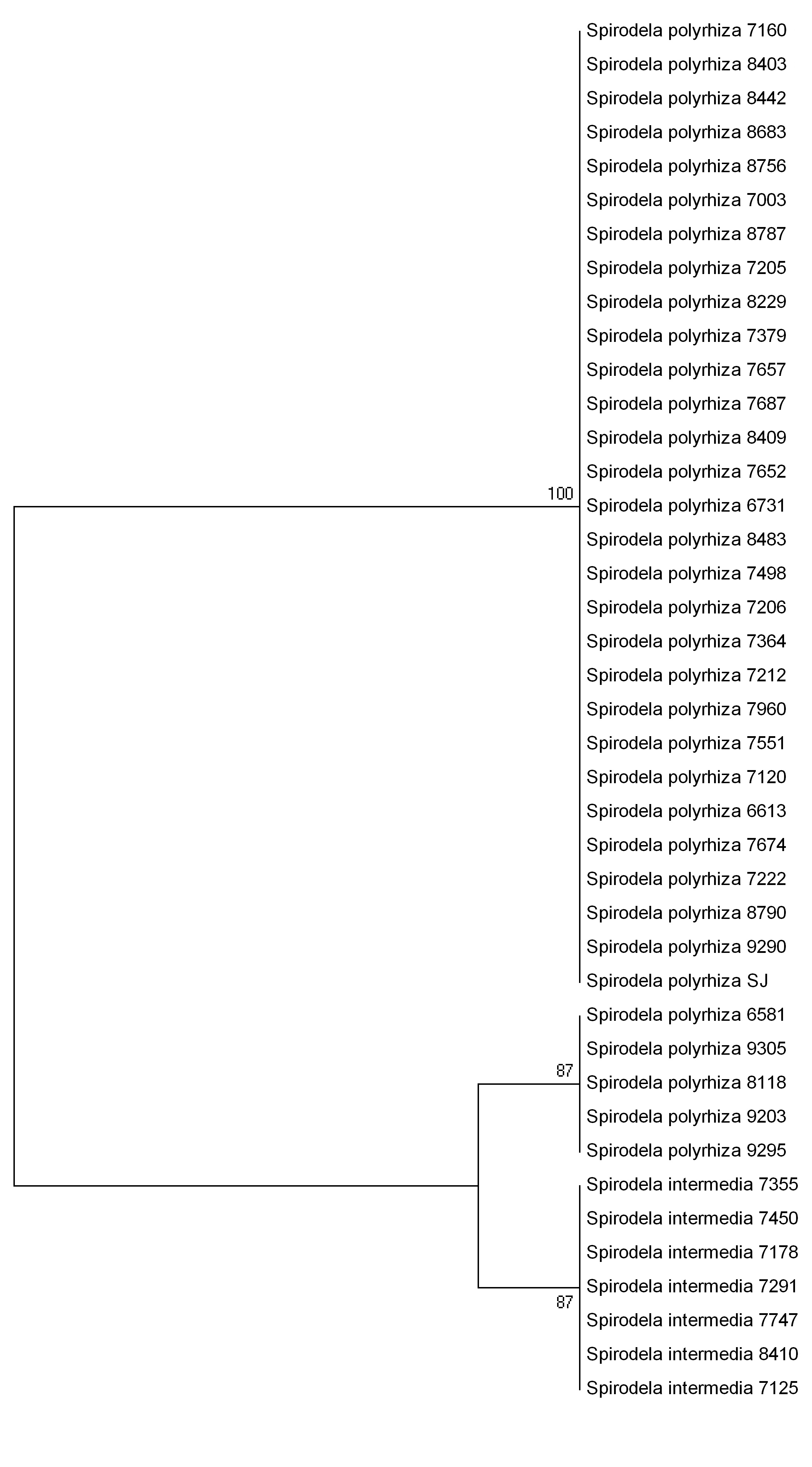

Supplement: Additional file 4 — UPGMA tree based atpF-atpH sequences for sister species of S. polyrhiza and S. intermedia. Distance analysis was carried out as described under Methods. [file 1471-2229-10-205-S4.TIFF]
